# Effect of Increasing Species Diversity and Grazing Management on Pasture Productivity, Animal Performance, and Soil Carbon Sequestration of Re-Established Pasture in Canadian Prairie

**DOI:** 10.3390/ani9040127

**Published:** 2019-03-29

**Authors:** Aklilu W. Alemu, Roland Kröbel, Brian G. McConkey, Alan D. Iwaasa

**Affiliations:** 1 Lethbridge Research and Development Centre, Agriculture and Agri-Food Canada, Lethbridge, AB T1J 4B1, Canada; akliluwake@yahoo.com (A.W.A.); roland.kroebel@canada.ca (R.K.); 2 Swift Current Research and Development Centre, Agriculture and Agri-Food Canada, Swift Current, SK S9H 3X2, Canada; brian.mcconkey@canada.ca

**Keywords:** Canada, grazing management, pasture mixture, re-established pasture, soil carbon

## Abstract

**Simple Summary:**

Canadian grasslands are recognized for providing high quality forage for grazing livestock and wildlife. The study was conducted on a re-established pasture in a Western Canadian semi-arid climate to investigate the effect of pasture species mixture and grazing management on pasture productivity, animal performance, and soil carbon sequestration. Pasture productivity and animal response were independent of pasture mixture but affected by grazing management. Average pasture dry matter productivity was greater with deferred-rotational grazing while pasture quality and animal gain were higher with continuous grazing. Soil carbon change varied with pasture seed mixture and grazing management interaction where pasture with 7-species mixture under continuous grazing had the lowest soil carbon gain.

**Abstract:**

The objective of the study was to determine the effect of type of pasture mix and grazing management on pasture productivity, animal response and soil organic carbon (SOC) level. Pasture was established in 2001 on 16 paddocks of 2.1 ha that had been primarily in wheat and summer fallow. Treatments consisted of a completely randomized experimental design with two replicates: two pasture mixes (7-species (7-mix) and 12-species (12-mix)) and two grazing systems (continuous grazing (CG) and deferred-rotational grazing (DRG)). Pasture was stocked with commercial yearling Angus steers (Bos Taurus, 354 ± 13 kg) between 2005 and 2014. All pastures were grazed to an average utilization rate of 50% (40% to 60%). Average peak and pre-grazing pasture dry matter (DM) yield and animal response were independent of pasture seed mixture but varied with grazing management and production year. Average peak DM yield was 26.4% higher (*p* = 0.0003) for pasture under DRG relative to CG (1301 kg ha^−1^). However, total digestible nutrient for pasture under DRG was 4% lower (*p* < 0.0001) as compared to CG (60.2%). Average daily weight gain was 18% higher (*p* = 0.017) for CG than DRG (0.81 kg d^−1^), likely related to higher pasture quality under CG. Soil carbon sequestration was affected by seed mixture × grazing system interaction (*p* ≤ 0.004). Over the fourteen years of production, pasture with 7-mix under CG had the lowest (*p* < 0.01) average SOC stock at 15 cm (24.5 Mg ha^−1^) and 30 cm depth (42.3 Mg ha^−1^). Overall, the results from our study implied that increasing species diversity for pasture managed under CG may increase SOC gain while improving animal productivity.

## 1. Introduction

Native prairie grasslands are recognized as important resources in producing quality forage for Canadian beef production. About 96% of the remaining 11.4 million ha of the native grassland and rangeland are used for grazing by livestock and wildlife [1]. Clayton et al. [2] indicated that prior to their conversion into cropland, it is estimated that about 61.5 million ha of Canadian prairie soils were covered by native grassland vegetation. Given the diverse and essential ecological resources and services provided by native grasslands [3,4], there have been, over the past decades, several federal and provincial initiatives in Canada to revegetate marginal annual croplands to perennial forage production [5,6,7]. Restoration and maintenance of native prairie grasslands can also provide an opportunity to mitigate greenhouse gas (GHG) emissions through soil organic carbon (SOC) sequestration [8,9,10]. Furthermore, management practices (e.g., grazing, burning, and fertilization) have been shown to influence carbon (C) sequestration of rangeland [8,10]. Therefore, improved grassland management practices that increase net accumulation of C in grasslands are gaining attention for their potential to minimize the rising concentration of atmospheric carbon dioxide. 

In Western Canada, forage species for grazing and hay production are predominantly seeded as either monocultures of grass or legume or as a binary grass-legume mix [11]. However, pasture seeded to a diverse seed mixture is considered as a comparatively productive and moreover more sustainable option [12,13]. Several ecological studies reported that plant species diversity/richness could: increase plant community stability and dry matter (DM) yield [13,14,15,16], contribute to greater forage intake by grazing animals [17], increase milk production [16,18,19], affect nitrogen excretion [20], improve root mass and soil fertility [14], have resilience to weed incursion [21], and increase SOC sequestration [22,23]. In addition to the positive association between annual DM yield and the number of plant species planted [13,24,25], Tilman et al. [26] and Schellenberg et al. [6] reported that pasture with species mixture of differing maturities (e.g., cool- and warm-season plant species) have the potential to provide higher quality of forage for an extended grazing season. However, others [16,27,28] questioned the lack of consistent benefit from increased plant diversity and the challenges in managing diverse species. This implies that there is a knowledge gap in how the concept of species diversity relate to managed and grazed pasture ecosystems. 

Selection of proper grazing management is important to attain economic benefits while maintaining the health of pastureland and grazing animals. For example, Conant et al. [10] reported that soil C sequestration can be increased on average by 0.28 Mg ha^−1^ year^−1^ following improved grazing practices (including lower stocking rates, seasonal grazing and rotational or short-duration grazing) and by 0.87 Mg ha^−1^ year^−1^ after conversion of cropland into pastureland. For Canadian beef production systems, a recent survey [29] indicated that 66% of the beef producers are using continuous grazing on native pastureland and the remaining 34% are using management-intensive rotational grazing. The benefits associated with the use of continuous or rotational grazing system have been a debate among researchers in the area [30,31]. Rotational grazing strategies have been promoted as a way to enhance vegetation and improve the sustainability of native grass-based pasture systems by increasing nutrient cycling [32]. However, ruminants are selective feeders and, thus, the performance of an individual can be higher under continuous than rotational grazing [32,33]. Others [34] reported no difference in animal performance (average daily gain, ADG) between short-duration and rotational grazing with comparable stocking rate. A review of rangeland studies [35] indicated mixed evidence with 92% of the studies reporting similar or greater individual animal responses with continuous than rotational grazing and 84% of the studies reporting similar or greater animal gain per unit land area with continuous compared to rotational grazing. Overall, the environmental and economic sustainability of each system depends on the management it receives [30,31]. 

The objectives of our study were to determine the effect of forage pasture mixture (7-species (7-mix) and 12-species (12-mix)) and grazing management (continuous grazing (CG) and deferred-rotational grazing (DRG)) on pasture productivity and quality, animal performance and SOC change in a Western Canadian semi-arid climate. We hypothesized that seeded native pasture with 12-mix would yield more herbage with better quality, better animal responses and greater SOC as compared to 7-mix under different grazing strategies.

## 2. Materials and Methods 

All experimental procedures were reviewed and approved by the Animal Care and Use Committee at the Agriculture and Agri-Food Canada, Swift Current Research and Development Centre (ACC 9850303) under the guidelines of the Canadian Council on Animal Care [36]. 

### 2.1. Study Site

The experiment was conducted between 2005 and 2014 at the Agriculture and Agri-Food Canada, Swift Current Research and Development Centre near Swift Current (lat. 50025’N, long. 107044’W; elevation 825 m), Saskatchewan, Canada. The soil was classified as a Swinton silt loam, an Orthic Brown Chernozem [37]. A completely randomized design was used with two replicates: two pasture seed mixtures (7-mix and 12-mix) and two different grazing systems: continuous grazing (CG) and deferred-rotational grazing (DRG). As details on pasture establishment and grazing management have been reported previously [5,6,38], only a brief description will be provided below.

### 2.2. Pasture Establishment

In spring of 2001, pasture was established on 33.6 ha of land (16 paddocks with 2.1 ha each) that was cropped since first cultivation in the 1920s. Seeding of native grass species (Table 1) was conducted on standing stubble, after any established weeds were removed by applying glyphosate the fall prior to seeding. The native grass seed mixture treatments consisted of a 7-species (7-mix) and 12-species (12-mix) mixture (Table 1). The 7-mix contained six species of native cool-season grass and one native legume species (purple prairie clover), whereas the 12-mix contained the 7-mix, plus an additional three species of native warm-season and two cool-season grasses. The seeding rate for the 7-mix and 12-mix treatments was 9.5 and 9.0 kg ha^−1^, respectively, seeded in combination with a disk drill with 22.5 cm row spacing at 0.6 cm depth. To avoid bridging in the seeder and foster initial growth, 18 and 34 kg ha^−1^ of 11-55-00 (N:P:K) fertilizer was applied in 2001 as a seed carrier for the 7-mix and 12-mix seed mixture, respectively. No fertilizer has been applied thereafter. 

**Figure A1:**
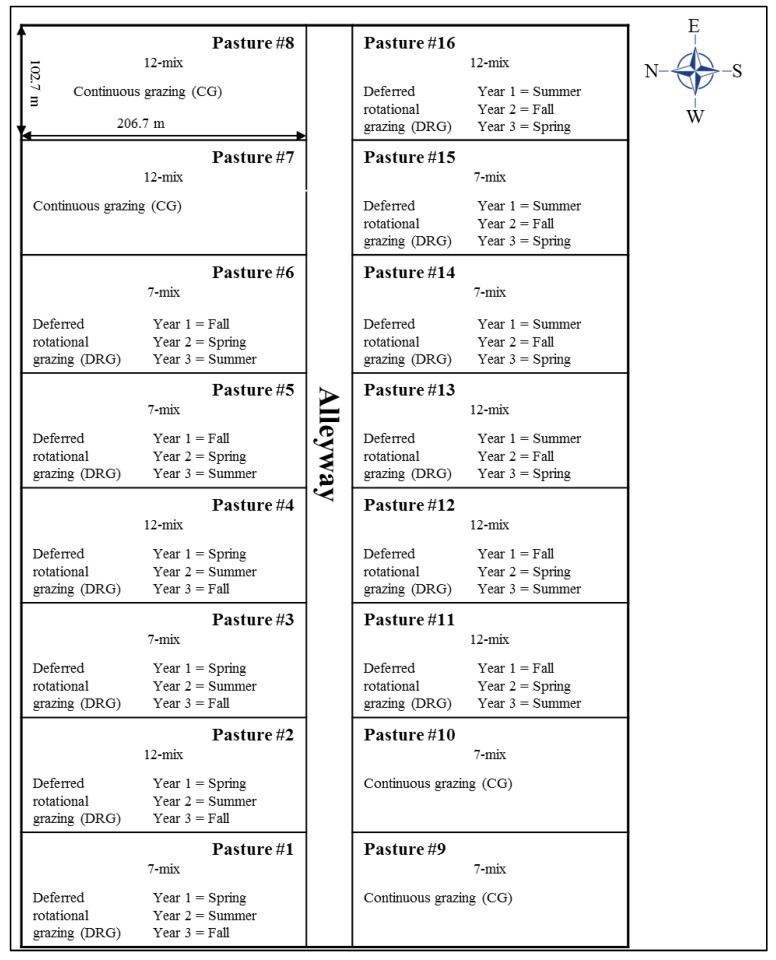
Pasture plots design of different grazing systems (continuous versus deferred rotational) on 7-mix or 12-mix mixed native grassland. The area of each pasture is 2.1 ha.

The sixteen paddocks were fenced after seeding in 2001. Between 2002 and 2004 another grazing study was conducted that investigated productivity and change in botanical composition of the pastures with different rates of utilization under continuous grazing [5,6]. The current study was conducted between 2005 and 2014. Pastures were stocked with commercial yearling Angus steers (Bos Taurus; 354 ± 13 kg). Four paddocks (two 7-mix and two 12-mix) were used for a CG system (Figure A1). There were three groups in the DRG system that started grazing at a different grazing season period (spring, summer, or fall) at the start of the study. Each group consisted of four pastures (two 7-mix and two 12-mix), and over the course of three years all three grazing season periods occurred for each pasture type. For example, for a paddock with grazing started in spring of 2004, summer of 2005, and fall of 2006, the second cycle of the grazing season period for that specific paddock started in spring of 2007. 

Weather data (monthly air temperature and rainfall) for the study period and long-term average at the experiment site were obtained from the weather station located near Swift Current, Canada (Table 2).

### 2.3. Animal and Grazing Managemnet

Grazing period started in June and ended by August for CG and lasted until the end of September for DRG. This corresponds to the common grazing season in southern Saskatchewan that extended between June and end of September (90 to 120 days long). For both grazing systems, average pasture utilization rate was maintained at an average of 50% (40 to 60%) which allows for all pasture plant species (i.e., cool and warm season grasses) to be grazed. According to Adams et al. [39], this is the ecologically sustainable rate of utilization that allows carryover of biomass to maintain other ecological functions. The number of animals on pasture and the number of grazing days were adjusted to maintain this utilization rate, and was calculated as ((pre-grazing forage yield-residual forage yield after grazing)/pre-grazing forage yield) ×100. Yearling steers were blocked by body weight and placed on each CG and DRG pastures. Steers were weighed after a 12 h fast at the start and end of the grazing seasons to calculate ADG. Total live weight production per unit area (ha) was calculated as ADG (kg ha^−1^) times grazing days (per ha) with grazing days equal to (total number of animals × days on test)/pasture area (ha).

### 2.4. Sample Collection and Analyses

Each year, four movable pasture cages (1 × 1.5 m) were randomly distributed on each pasture prior to the commencement of grazing (total of 64 cages) to measure pasture forage yield and quality for the grazing season [40]. Pre-grazing pasture DM yield (end of June) was estimated according to the procedure of Cook and Stubbendick [40] by taking ten representative 0.25 m^2^ quadrat samples (ten per pasture) randomly throughout the pasture. For estimation of maximum/peak forage DM yield and pasture quality, plants in the 0.25 m^2^ area inside the four cages were hand clipped to a height of 2.5 cm above the soil surface at the end of July. At the beginning of the establishment of the different pastures, the native and weed plant material was separated for each sample and plant materials were dried (forced air oven) for 48 h at 60 °C for DM determination. However, after a few years the native stands were established and no separation of the collected sample was needed. At the end of the grazing season after the steers were removed, residual pasture yield for each grazed field was determined using 0.25 m^2^ quadrat square to estimate the utilization rate. In addition to the movable cages, a permanent grazing enclosure (3.6 × 3.6 m) was located near the center of each pasture that was excluded from grazing. 

Samples for plant biomass and litter (all standing and fallen dead materials) were collected in the fall of 2004, 2008, 2011, and 2014 (at the same time as soil sampling). Three random samples were collected, two from areas adjacent to grazing enclosures and one from the inside of one of the four grazing enclosures. All vegetation within a 0.25 m^2^ quadrat was clipped at the ground level and the surface soil was raked with a hand-fork to remove litter above the soil mineral surface. All material collected was separated into live (any green material) and dead components (litter) and washed to remove any soil material before oven-dried (60 °C for 48 h) for further analysis. 

For all treatments, sub-samples of native plants taken from each grazing season forage yield analysis were ground through a 1-mm screen Wiley mill (Thomas-Wiley, Philadelphia, PA) and analyzed for organic matter (OM), OM digestibility (OMD), crude protein (CP), neutral detergent fiber (NDF), acid detergent fiber (ADF) and mineral (total potassium). Neutral detergent fiber and ADF were determined as described by Van Soest [41] using the ANKOM 2000 fiber analyzer (ANKOM Technology, Fairport, NY) with the addition of sodium sulfate and α-amylase for NDF. Nitrogen (N) fraction in native plant vegetation and litter samples was determined using the Kjeldahl digestion method [42]. The total Kjeldahl N was multiplied by 6.25 to determine the level of CP. Carbon fraction was determined using the Leco Carbon Determinator [43]. 

In the fall of 2000, soil samples from all the sixteen pastures were collected from five different locations and these sites were permanently marked for future sampling. At each site location, core samples (6.3 cm cutting edge diameter) samples were taken at 0–15 cm and 15–30 cm depth, respectively. Prior to sampling, all residues from the sampling spot were cut and removed. Analysis of soil samples taken in 2000 indicated small variation within pastures and therefore, only three of the five sites within each pasture were subsequently sampled. As such, during 2004, 2008, 2011, and 2014 sampling years, soil sample from three micro-sites within each pasture were taken and core samples from each soil depth were pooled for analysis. All soil samples were bagged and initially stored at 2 °C until further analysis. 

Soil samples were analyzed for soil moisture, bulk density (BD), organic C and N using the Methods Manual Scientific Support Section Analytical Chemistry Laboratory [44]. Soil bulk density was determined for individual sample based on the fresh gross soil weight and moisture content. Soil C stock was calculated as a product of the measured C (%), BD (g cm^−3^) and soil depth (cm) for each sample. However, to compare management-induced changes in SOC, it is generally considered appropriate to express C as an equivalent soil mass [45,46]. We therefore calculated SOC and N using equivalent mass approach following Ellert and Bettany [45] and Ellert et al. [46] to attain an equivalent soil mass of 4000 Mg ha^−1^ at a 30 cm sampling depth.

### 2.5. Statisitcal Analysis

Data was analyzed as a 2 × 2 factorial design in a randomized complete block design with two replications using SAS 9.3 Proc Mixed Model [47] to determine the effect of seed mixture (7-mix and 12-mix), grazing system (CG and DRG), production year and their interaction on pasture productivity (maximum/peak and pre-grazing forage DM yield, forage biomass, litter), forage quality (OMD, NDF, ADF, CP, DE), animal response (ADG, grazing/animal days, total live weight production, stocking rate) and soil characteristics (C, N, BD, moisture). The model included the effect of seed mix, grazing system, year and their interaction as a fixed effect and replication as a random effect. For DRG, the effect of start of grazing season periods (spring, summer, or fall) was tested but the effect was not significant and hence excluded from the model. Sub-samples from each pasture were averaged and mean values were used for analysis. Residual plots were used to check the validity of the underlying statistical assumptions of homogeneity of variances and normality. Univariate procedure was used to determine if the difference of mean estimates was different from zero (*p* ≤ 0.05). Tendency for significance was determined at *p* < 0.10. A simple correlation analysis was conducted to determine the relationship between climate variables (precipitation and temperature) and pasture productivity and quality and SOC.

## 3. Results

### 3.1. Climatic Condition

Growing conditions varied over the nine years study period (2005 to 2014; Table 2). While the mean May and June temperature was similar to the long-term average throughout the experimental period, the mean March temperature was 5.4 °C in 2010 and 5.9 °C in 2012 above the long-term average (–4.2 °C). Furthermore, the July temperature in 2007 was 4.2 °C above the long-term average (18.7 °C). Annual mean temperature was 0.1 °C to 0.7 °C below long-term average in 2009, 2013, and 2014 but 0.1 °C to 3 °C above average in 2005, 2006, 2007, and 2012. Average growing season (April–October) temperature was 1.0 °C (2006), 0.9 °C (2007) and 0.4 °C (2012) higher than the long-term average for the season (12.2 °C). The mean July and August rainfall in 2007 was 20–40 mm lower as compared to the long-term average. However, the mean May and June rainfall in 2010 was 52–67 mm higher than the long-term average. Annual mean rainfall was also varied among the experimental year, lower than long-term average (367 mm) in 2007 and 2009 but higher than long-term average in 2008, 2010, 2011, and 2014 (Table 2). Growing season (April–October) rainfall in 2010 was 50% higher than the long-term average (287 mm) for the season.

### 3.2. Effcet of Seed Mixture 

The effect of seed mixture on pasture productivity and cattle performance and pasture quality is shown in Table 3 and Table 4, respectively. Maximum/peak, pre-grazing pasture and animal response (ADG and total live weight production per unit area) were independent of pasture seed mixture. However, there was a trend (*p* ≤ 0.06) for an interaction of seed mixture and grazing system for maximum/peak and pre-grazing forage DM yield and stocking rate. Average pre-grazing pasture DM yield was higher (*p* = 0.057) for the 12-mix under DRG (1496 kg ha^−1^) and lower for the 12-mix under CG (756 kg ha^−1^; Table 3). Furthermore, there was no interaction effect between pasture seed mixture and production year on peak and pre-grazing pasture DM yield (Table 3). However, pasture DM yield varied (*p* ≤ 0.002) over the experimental year with the lowest pre-grazing pasture biomass yield observed in 2013 (737.2 kg DM ha^−1^) and 2014 (780.4 kg DM ha^−1^). Pasture seed mixture had no effect on animal responses: ADG (*p* = 0.75), number of grazing/animal days per ha (*p* = 0.12) and total live weight produced per unit area (*p* = 0.19; Table 3). However, ADG and total live weight per ha varied (*p* ≤ 0.001) among the experimental year, which could be related to the observed variations in forage DM yield and quality.

**Figure A2:**
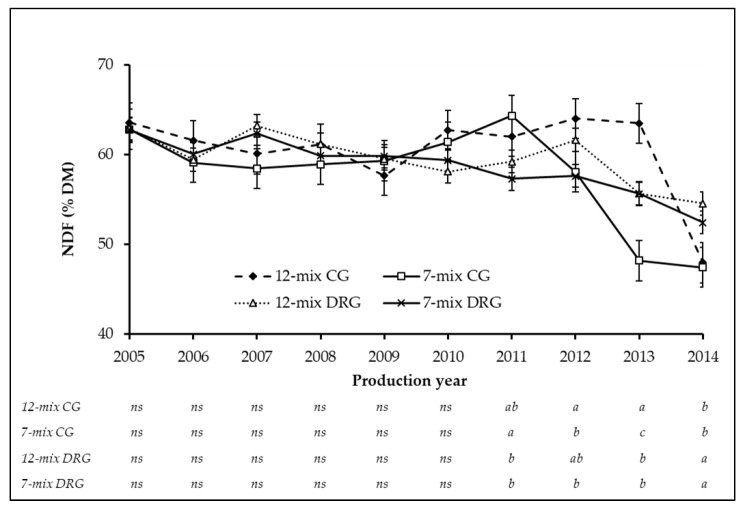
Pasture NDF content (%DM) for the seed mixture (7-mix, 12-mix) × grazing system (CG = continuous grazing, DRG = deferred rotational) × year interaction (*p* < 0.05). Significance difference among treatment within a year was indicated by lower case letters (*a*, *b*, *c* and *ns* = not significant) and standard error of means was indicated by error bars.

With regard to pasture forage quality, seed mixture had no effect on OMD and CP (Table 4). However, TDN (related to pasture ADF) tended to be higher (*p* = 0.065) for pasture with 7-mix (59.5%) than 12-mix (58.5%). Total pasture phosphorus content varied (*p* ≤ 0.03) between seed mixture with the 7-mix having 13.3% higher total phosphorus as compared with 12-mix (1.5 g kg^−1^ DM). A three-way interaction (seed mix × grazing system × year, *p* = 0.03) was observed for pasture NDF (Table 4) where the highest value (64.4% DM) was observed for the 7-mix under CG in 2011 and the lowest (47.6% DM) for 7- and 12-mix under CG in 2014 (Figure A2). 

### 3.3. Effcet of Grazing Managemnet

**Figure 1:**
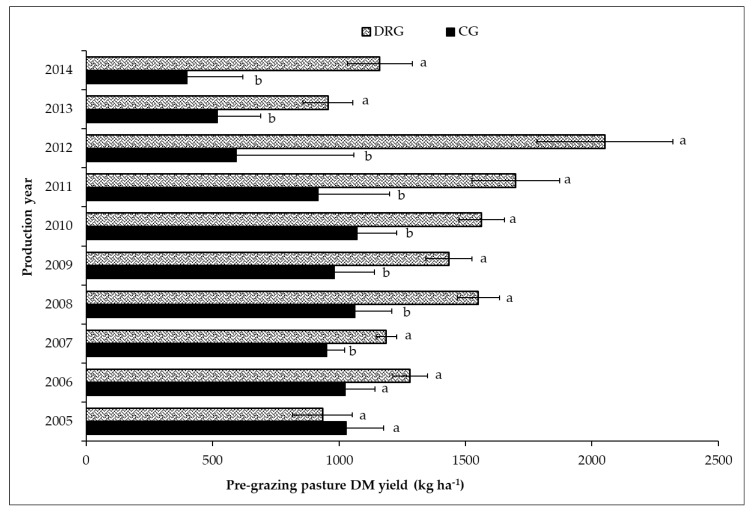
Pre-grazing pasture biomass yield (kg ha^−1^) harvested at the end of June for the grazing system (CG = continuous grazing, DRG = deferred rotational) × year interaction (*p* ≤ 0.05) over the experimental period. Significance difference among treatment within a year was indicated by lower case letters (a, b) and standard error of means was indicated by error bars.

Pasture productivity, pasture quality, and animal responses were influenced by grazing management strategies over the study period (Table 3 and Table 4). Maximum/peak DM yield were varied between grazing systems where DRG had higher (*p* = 0.0003) maximum/peak (1644 kg ha^−1^) relative to CG (1301 kg ha^−1^). Significant (*p* = 0.006) grazing system by year interaction was observed for pre-grazing forage DM yield and stocking rate (Table 3, Figure 1). The highest average pre-grazing forage DM yield was observed with DRG in 2012 (2051.8 kg ha^−1^) and the lowest was with CG in 2014 (399.2 kg ha^−1^; Figure 1). The number of grazing days per unit area was higher (*p* = 0.05) for pasture under DRG (63.4) relative to CG (56.2). 

Grazing system and production year affected forage quality (Table 4). Due to the lower (*p* < 0.001) forage ADF content for pastures under CG (34.4 vs. 36.2% DM), OMD (53.7 vs. 50.3%), TDN (60.2 vs. 57.8%), and DE (11.1 vs. 10.6 MJ kg^−1^ DM) were greater (*p* < 0.0001) for CG as compared to pasture managed under DRG. Pasture CP tended (*p* = 0.065) to be higher for pasture under CG (6.8% DM) than under DRG (6.4% DM). The observed variation in pasture productivity and quality between grazing systems influenced animal responses (Table 3). Average daily gain was 21.6% higher (*p* = 0.02) for CG as compared to DRG (0.81 kg d^−1^; Table 3). However, despite the differences in ADG, total live weight production per unit area was similar (*p* = 0.525) between grazing systems, 56.0 kg ha^−1^ for CG and 52.7 kg ha^−1^ for DRG (Table 3).

### 3.4. Soil Organic Carbon and Above Ground Biomass and Litter Composition

Pasture above ground total biomass and litter production and its respective C and N content were not affected either by pasture seed mixture or the interaction between seed mixture and grazing system (Table 5). However, biomass C:N was 15% higher (*p* = 0.045) for pasture under DRG as compared to CG (59.3). Similarly, pastures under DRG had 36% higher litter production (*p* = 0.018) relative to pastures managed under CG (188 kg ha^−1^), which affected the amount of litter C and N produced per ha.

Over the production year, significant variation (*p* ≤ 0.001) in average biomass and litter production (kg ha^−1^) and its C and N composition were observed (Table 5). The highest mean above ground biomass production (851 kg ha^−1^) was observed in 2014, whereas litter production (329 kg ha^−1^) was greater in 2004. In terms of variations in C:N ratio, the lowest value was observed in 2004 for above ground biomass (45.4) and for litter in 2008 (43.1; Table 5).

Soil C stock calculated on a standard 15 cm and 30 cm soil depth was affected (*p* ≤ 0.004) by seed mixture and grazing system interaction (Table 6). For the top 15 cm soil depth, the lowest SOC was observed for the 7-mix under CG (24.5 Mg C ha^−1^) and the highest for 12-mix managed under the same grazing system (31.0 Mg C ha^−1^). Although the observed difference in SOC for the 15 cm depth had no impact on soil BD, soil moisture was higher (*p* = 0.031) for the 7-mix under CG (Table 6). Similarly, for the 30 cm soil depth, 7-mix pasture under CG had the lowest C stock (42.33 Mg C ha^−1^) relative to the other treatments. Soil moisture at the 30 cm soil depth varied (*p* < 0.026) between seed mixture where the 7-mix pasture had 6.3% higher moisture content relative to 12-mix pasture (14.4%; Table 6).

Soil organic C, moisture, BD and C:N ratio at both sampling depths were varied (*p* ≤ 0.005) among production year but there was no two- or three-way interaction observed with seed mixture and grazing system (Table 6). For the 15 cm soil depth, the highest SOC values were observed in 2004 (30.97 Mg ha^−1^) and 2014 (30.81 Mg ha^−1^). These values were 11.6 to 12.2% higher (*p* ≤ 0.05) than the initial SOC value in 2000 (27.61 Mg ha^−1^; Table 6). Similarly, for the 30 cm depth, the highest average C stock was observed in 2014 and was 10.3% higher (*p* = 0.043) than the initial estimate in 2000 (46.6 Mg ha^−1^). Over the 14 years of production, average annual soil C sequestration were 0.23 Mg C ha^−1^ year^−1^ (ranging from 0.11 to 0.25) for 15 cm depth and 0.34 Mg C ha^−1^ year^−1^ (ranging from 0.22 and 0.51) for 30 cm depth. 

**Figure 2:**
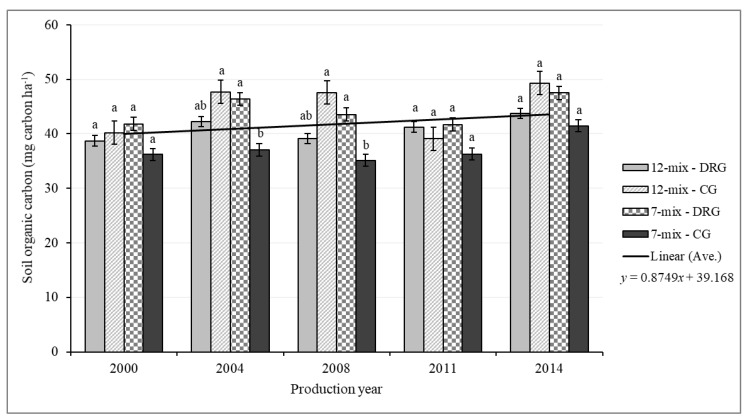
Soil organic carbon stock adjusted for equivalent soil mass of 4000 Mg ha^−1^ within a 30 cm depth for the seed mixture (7-mix, 12-mix) × grazing system (CG = continuous grazing, DRG = deferred rotational grazing) interaction within the production year of 2000 to 2014. Significance difference among treatment within a year was indicated by lower case letters (*a, b*) and standard error of the means was represented by error bars.

When soil C stock results were corrected for equivalent mass, significant seed mix by grazing system interaction (*p* = 0.012) and production year (*p* = 0.04) effects were again found (Table 6; Figure 2). Correction for the equivalent mass had an effect of merely enhancing the differences among the management scenarios. Average annual soil C sequestration was 0.45 Mg C ha^−1^ year^−1^, ranging between 0.36 Mg C ha^−1^ year^−1^ and 0.65 Mg C ha^−1^ year^−1^. Overall, an increasing trend in SOC over the experimental year was observed for the 30 cm sampling depth with the 7-mix under CG had numerically lower estimates throughout the sampling year (Figure 2).

## 4. Discussion

The effect of climatic factors (e.g., temperature, precipitation) on pasture composition, species co-existence, productivity and quality has been reported in several previous studies [50,51,52,53,54]. Ren et al. [54] reported that pasture vegetation dynamics and species co-existence can be determined by temporal variability in precipitation and temperature rather than grazing management. Furthermore, Paruelo and Lauenroth [50] reported that temperature, precipitation, and seasonal distribution of precipitation are the primary factors that affect the relative aboveground productivity of cool- and warm-season grasses and shrubs. Over the course of the nine years of our study (2005–2014), severe drought (2007 and 2009), excess precipitation (2010), hot and dry summer (2012) and longer winter and early fall seasons (2013) were observed (Table 2). This contributed to the observed differences in pasture composition, productivity and quality. For example, the lowest pre-grazing pasture DM yield observed in 2013 and 2014 (Table 3) could be related to the long winter and early fall seasons observed in 2013. A simple correlation analysis indicated that spring precipitation (April + May + June) explained about 22% of the variation in available pasture yield whereas spring temperature (April + May + June) accounted for 51% of the variation in maximum/peak forage yield. Using data from a long-term (16 years) grazing study on pasture managed under different grazing system (short-duration or season-long) and stocking rate (light, moderate and heavy), Derner and Hart [51] reported that spring precipitation could explain 54 to 67% of the variation in maximum/peak forage yield. This implies that climatic factors play a major role in pasture management.

### 4.1. Effcet of Seed Mixture

Pre-grazing pasture DM yield and forage quality values for 7-mix and 12-mix were comparable with previous reports by Iwaasa et al. [5] and Schellenberg et al. [6]. The lack of clear effect between pasture mix on pasture productivity in our study was in line with previous reports [16,24,26,55,56]. For example, Tilman et al. [26] and Deak et al. [56] reported greater yield with increased species richness. In contrast, other studies [13,57,58] showed that DM production is independent of mixture complexity (number of species) but rather depends on the contribution and type of individual species in the mixture (composition). This is because DM yield is a function of particular species that composed the mixtures rather than the mixture complexity [16,57]. Furthermore, a weak relationship (r^2^ = 0.20) between the number of species in the seed mixture and herbage yield has been reported by Sanderson et al. [13] for mob-grazed pasture. Using mob-grazing, Deak et al. [56] concluded that forage yield and quality are greatly influenced by the individual species in the mixture than mixture complexity. In our study, the 12-mix pasture had higher plant diversity or complexity due to the inclusion of warm-season grasses (C_4_) that contributed about 8.6% in 2004 and 28.3% in 2014 (Table A1). However, most of the C_4_ grass contribution (24.8%) was by Little Bluestem (Schizachyrium scoparium (Michx.) Nash). Furthermore, 57% (in 12-mix) to 76% (in 7-mix) of the total foliar contribution was by others including moss, litter, weed and bare ground/dung which may contributed for the lack of difference in DM yield between the two pasture seed mixtures. 

Pasture fiber concentration (NDF, ADF), which influences digestibility was higher for 12-mix than 7-mix pasture and varied among the production year (Table 4). The observed differences in pasture quality could be due to the inclusion of the three C_4_ grasses (Little Bluestem (Schizachyrium scoparium (Michx.) Nash), Blue grama (Bouteloua gracilis (Willd. ex Kunth) Lag. ex Griffiths) and Prairie sandreed (Calamovilfa longifolia (Hook.) Scribn.)) in the 12-mix pasture and its increased foliar contribution. Warm-season grasses use the C_4_ metabolic pathway for photorespiration whereas the cool-season (C_3_) grasses use C_3_ carbon fixation [58]. Often the C_4_ metabolic pathway leads to a higher rate and degree of deposition of lignin in the plant tissue that affects nutritional quality and digestibility [59,60,61]. It has been reported that warm-season grasses have lower CP, higher fiber, and lower digestibility as compared to cool-season grasses [59,61,62]. This is supported by Archimède et al. [61], who conducted a meta-analysis and reported that on average C_4_ grasses in the database had about 16% higher NDF content than C_3_ grasses (64.6 vs. 55.7%, respectively). Furthermore, the proportional foliar cover contribution of warm-season grass in the 12-mix increased over time (Table A1).

Generally, the effect of pasture mixture on animal performance is not well studied. Pasture seed mixture had no effect on animal weight gain and total live weigh per unit area in the current study. Some authors [63,64] speculated that cattle grazed on pasture with diverse mixture may exhibit better performance since they have the opportunity to select from a variety of forage plants while others reported no effect of pasture mixture on animal performance [21,65,66,67]. In the current study, stocking rate was similar between pasture mixtures (3.6 vs. 3.7 AUM ha^−1^; Table 3) and pasture utilization rate was maintained at moderate level (53–54%), which minimizes selection and allows carryover of biomass to maintain other ecological functions [39]. Using 3-years of grazing data, Tracy and Faulkner [21] reported no effect of pasture species richness on daily weight gain and gain per ha for beef cows and calves rotationally managed on pastures with three (simple), five (medium) and eight (complex) species mixtures containing tall fescue (Lolium arundinaceum (Schreb.) S.J. Darbyshire), orchardgrass (Dactylis glomerata L.) and white clover (Trifolium repens L.) as common domain. In their study, grazing management (e.g., stocking rate) and climatic effects on forage availability appeared to be more important in affecting cattle performance. Similarly, Wedin et al. [65] reported no benefit to planting a complex mixture of grass and legumes for grazing on carrying capacity and milk production. Further study is warranted to investigate the relationship between pasture species diversity and animal performance. 

### 4.2. Effcet of Grazing Managemnet

The effect of grazing management on pasture composition, productivity and quality and animal response has been well documented [6,31,32,33,34,35,68,69,70]. However, benefits related to pasture and animal from continuous and rotational grazing systems has long been debated [31,33,34]. Grazing disturbance generally has been reported to be beneficial for maintaining species diversity [6,68] and improving above ground production through increased tillering and rhizome production [69,70]. Generally, selection of proper grazing management is important to ensure productivity, sustainability and animal health, which in turn impacts cost of production. 

The impact of grazing system on pasture productivity and quality could relate to its influence on plant height and maturity since the physiological age of plant tissue is a major factor affecting forage quality [71]. Pasture managed under CG system has shorter plant height with lower fiber (ADF, NDF) relative to rotational grazing with longer, more mature plants and dead tissues [72]. Acid detergent fiber is an index of digestibility and its concentration increases with plant maturity [73]. In our study, ADF concentration was 5% lower (*p* < 0.001) and OMD was 6% higher (*p* < 0.001) for pasture under CG than under DRG, 36.2% DM and 50.3%, respectively (Table 4). The lower OMD in DRG could also be related to the higher accumulation of dead plant materials (litter; Table 5) because OMD is higher in live tissues than dead senesced plant tissues [71]. 

Previous studies reported no substantial increases in livestock productivity with the use of deferred-rotational systems as compared to CG, regardless of the lower pasture productivity with CG system [35,74,75,76]. Research conducted in the Kansas Flint Hills reported a 17% higher herbage production for a 3-pasture, deferred-rotation system but steers on a season-long, continuously grazed pasture weigh 10.4 kg heavier at the end of the grazing season [74]. Similarly, although maximum/peak and pre-grazing pasture DM yield were greater for DRG, ADG was 18% higher for CG (0.8 vs. 1.0 kg d^−1^; *p* = 0.017), and there was no difference in total live weight production per unit ha between the two grazing systems. The higher ADG for CG may, in part, be due to lower stocking rate and consumption of high-quality forage, high OMD and DE and low ADF concentration (Table 4). In the DRG system animals had to eat low quality, mature forage whereas in CG system, animals could graze on high quality regrowth from previously grazed plants. Overall, this implies that higher pre-grazing standing forage does not necessarily indicate higher livestock production per unit area but the more herbage production in the DRG allows increased stocking rate and better long-term survival of native range [1]. 

The different stocking rates used in DRG (4.5 AUM ha^−1^) and CG (2.5 AUM ha^−1^) could have a confounding effect for the difference in pasture productivity and quality. Previous reports [77,78] have shown that stocking rate is a confounding factor on grazing system effect on animal weight gain. Often, higher stocking rate is used in rotational grazing, and stocking rate has been shown to be one of the major factors that influence cattle performance through its impact on forage availability and quality [79,80]. In support of this generalization, several grazing studies under different environmental conditions evaluated continuous and rotational grazing using equivalent stocking rate and reported no difference in pasture productivity and quality and animal performance [35,78,81,82]. Pitts and Bryant [81] used equal stocking rate for continuous and rotational grazing and reported no differences in pasture productivity and daily weight gain but when stocking rate doubled for rotational grazing, animal daily gain reduced by 40% relative to continuous grazing. For continuous and rotational grazing systems under similar stocking rate, Manley et al. [82] reported no effect of grazing management and a reduction in weight gain per head with increasing stocking rate for both grazing systems. Furthermore, animal performance has been shown to decrease as stocking rate increases [33].

### 4.3. Soil Carbon Sequestration 

The average SOC at the start of the study in 2000, prior to seeding of the native pasture, was 27.6 and 46.6 Mg ha^−1^ for the 15 cm and 30 cm depths, respectively, which were expected for the land that had been in a crop-fallow rotation system for more than 80 years [83]. The average C sequestered over the 14 years of production was 3.20 Mg ha^−1^ for 15 cm depth and 4.82 Mg ha^−1^ for 30 cm depth with average annual rates of 0.23 Mg ha^−1^ for 15 cm and 0.34 Mg C ha^−1^ year^−1^ for 30 cm (Table 6). These values were within the range of previously reported soil C sequestration estimates when cropland converted into grasslands. Annual soil C sequestration rate can range between 0.1 and 0.87 Mg C ha^−1^ year^−1^ when cropland converted into pasture (tame and native forage species), and the highest sequestration rate are achieved within the first 10 years for established perennial grassland after seeding [10,72,84,85]. Russell and Bisinger [86] argued that due to the high C variability, more than 10 years is needed to accurately detect the C sequestration benefits from management. In agreement, within the 14-years of this study we observed significant variability in soil C stock between sampled years (Figure 2), which indicates that change in soil C for land-use change has not yet equilibrated to a new steady-state value. Furthermore, the average annual soil C sequestration rate can be affected by soil type, grass mixtures and grazing systems [9,22]. For Canadian grasslands in black, brown and dark brown soil zones, Wang et al. [9] compiled previous long-term studies and reported a net C sequestration of 0.22 Mg ha^−1^ year^−1^, 0.14 Mg ha^−1^ year^−1^, and 0.12 Mg ha^−1^ year^−1^, respectively, in the top 15 cm depth. However, in East Central Saskatchewan (thin Black and dark Brown soils), Mensah et al. [87] reported a higher net C gain (0.6 Mg ha^−1^ year^−1^ to 0.8 Mg ha^−1^ year^−1^ in 15 cm) for a seeded grassland on formerly cultivated land under light grazing or haying within 5–12 years after establishment using a mixture of wheatgrasses (Agropyron spp), blue grama grass (Bouteloua gracilis) and alfalfa (Medicago sativa). Our study was conducted in a brown soil zone of Canadian prairie [35] and also used native grass seed mixture. Varying level of soil C sequestration have also been reported for short and mixed grasses in the Northern Great Plains using long-term grazing data. For example, using a grazing management data collected over 44 years (1959 to 2003) in the northern mixed-grass prairie, Liebig et al. [88] reported an annual C gain rate of 0.39 and 0.41 Mg ha^−1^ year^−1^ for moderately and heavily grazed pasture, respectively. Similarly, for short to mixed grass prairie managed under different grazing systems in the United States, Derner and Schuman [89] reported a net C sequestration ranging from 0.07 Mg ha^−1^ year^−1^ to 0.30 Mg ha^−1^ year^−1^. 

Soil organic C was measured every three or four years and average gain in SOC stock was observed only in 2004 and 2014 since the conversion of cropland to pastureland in 2000 (Figure 2). The observed inconsistencies in soil C gain in our study could partly be related to the variation in precipitation and temperature (Table 2). We observed that about 28% of the variation in SOC gain was explained by precipitation adjusted for long-term average. Long-term studies of annual crop rotation systems in the semiarid Canadian prairie indicated that dependent on the amount and timing, increased (above-average) precipitation may favor SOC loss through increased decomposition more than SOC gain from increased via C inputs [90,91]. The loss of SOC in 2008 could be due to the increased decomposition rate resulting from the increased precipitation in 2008 relative to 2007 and the long-term average. For a nearby site, Maillard et al. [92] reported that SOC dropped dramatically in wet years. In addition to that, the drier condition in 2007 may affect the aboveground net primary production and as such, the litter C inputs into the soil (Table 5). Whereas, the abundant moisture in 2010 and 2011 may facilitate decomposition that resulted in a negative SOC balance due to increased SOC loss that exceeds the amount of C entering the system. Several studies also reported the impacts of pasture management and environmental factors on SOC stocks [89,93,94]. Using data from grass-based pastures in Southern Australia, Sanderman et al. [94] reported no difference in SOC (30 cm depth) between pasture managed under continuous and rotational grazing and 42 to 60% of the variation in the annual SOC gain can be explained by a long-term mean annual precipitation. Piñeiro et al. [93] investigated the pathways for the impact of grazing and non-grazing on SOC and found that SOC stock is higher under the driest and wettest environmental conditions but lower at intermediate precipitation (400–800 mm) for both management scenarios. 

Soil C was affected by seed mix × grazing system interaction where the 7-mix pasture under continuous grazing had the lowest average SOC stock both at the 15 cm depth (24.5 Mg ha^−1^) and in 30 cm depth (42.3 Mg ha^−1^; Table 6). The observed interaction effect could be due to the differences in the proportional foliar contribution of C_3_ and C_4_ grass species in the 7- and 12-mix pasture (Table A1) and the variation in the responses of C_3_ and C_4_ grass species to grazing management [22,95,96]. For the 12-mix pasture, the proportional contribution of C_4_ grasses increased from 8.6% in 2004 to 28.3% in 2014 while the proportion of C_3_ grasses reduced from 27.0% in 2004 to 14.8% in 2014. Mcsherry and Ritchie [22] reported that the interaction of grass species composition and grazing intensity is a significant biotic driver for the impact of grazing management on SOC. They noted that under moderate and heavy stocking rates, grazing has a positive impact on SOC for pasture dominated by C_4_ and mixed grasses and a negative impact on pasture dominated by C_3_ grasses. Similarly, in a shortgrass steppe and a northern mixed-grass prairie, Derner et al. [95] observed that moderate and heavy stocking rates reduced the proportion of C_3_ perennial grasses while increasing the predominant C_4_ perennial grass (blue grama). In our study, stocking rate tended to be influenced by pasture mixture and grazing system interaction (*p* = 0.06), which may have contributed to the observed variations in SOC gain. Variation in the responses of C_3_ and C_4_ grass species to grazing management has been reported (22), which may contributed to the observed interaction effect between seed mixture and grazing system on SOC. Derner et al. [95] and Frank et al. [96] implicated that the stimulation of the fine, shallow roots by grazing in C_4_ species may affect the belowground C allocation and soil C change. Furthermore, a higher root-to-shoot ratio [97], greater root density and turnover [96,98] and higher mycorrhizal association [99] have been reported for C_4_ grass species relative to C_3_ grasses. 

## 5. Conclusions

Our study showed that pasture yield and quality and cattle performance were independent of native grass species mixture but varied with grazing management (CG and DRG). The increased pre-grazing pasture DM yield for DRG resulted in a greater stocking density but reduced pasture quality and animal performance. Soil organic C stock (at 15 cm and 30 cm sampling depth) was the product of seed mixture and grazing system interaction with the lowest average stock was observed for the 7-mix pasture under CG. Overall, regardless of seed mixture and grazing system, SOC was increased annually by 0.23 Mg ha^−1^ and 0.34 Mg ha^−1^ for the 15 cm and 30 cm soil depth, respectively, over the 14 years period. The variation in SOC level over the production year was expected given the differences in environmental conditions experienced among the different soil sampling years. Continuous grazing is a common management practiced by most Canadian beef producer. Our study implied that for pasture under CG, increasing pasture species diversity may increase SOC gain while improving animal responses. For a better understanding of the impact of seed mixture, pasture management and their interaction on grassland ecosystem dynamics which include climate-plant-animal-soil-microbial interactions, continued multi-disciplinary long-term research is required.

## Figures and Tables

**Table 1:** Species name of the two native plant mixtures and seeding rate of each species.

Pasture Mixture	Mixture Species Name	Seeding Rate (PLS m^−2^) ^a^
7-mix (7 species)	Western wheatgrass (*Pascopyrum smithii* (Rydb.) Barkworth & D.R.) ^b^	14
	Northern wheatgrass (*Elymus macrourus* (Turcz. *ex* Steud.) Tzvelev) ^b^	14
	Awned wheatgrass (*Elymus trachycaulus* (Link) Gould & Shin. ssp *subsecundus* (Link) A.&D. Love.) ^b^	14
	Slender wheatgrass (*Elymus trachycaulus* (Link) Gould subsp. *Trachycaulus*) ^b^	14
	June grass (*Koeleria macrantha* (Ledeb.) Schult.) ^b^	14
	Green needle grass (*Nassella viridula* (Trin.) Barkworth) ^b^	14
	Purple prairie clover (*Dalea purpurea* Vent.) ^c^	14
12-mix (12 species)	Western wheatgrass (*Pascopyrum smithii* (Rydb.) Barkworth & D.R.) ^b^	8
	Northern wheatgrass (*Elymus macrourus* (Turcz. *ex* Steud.) Tzvelev) ^b^	8
	Awned wheatgrass (*Elymus trachycaulus* (Link) Gould & Shin. ssp *subsecundus* (Link) A.&D. Love.) ^b^	8
	Slender wheatgrass (*Elymus trachycaulus* (Link) Gould subsp. *trachycaulus*) ^b^	8
	June grass (*Koeleria macrantha* (Ledeb.) Schult.) ^b^	8
	Green needle grass (*Nassella viridula* (Trin.) Barkworth) ^b^	8
	Purple prairie clover (*Dalea purpurea* Vent.) ^c^	8
	Needle and thread grass (*Hesperostipa comata* (Trin. & Rupr.) Barkworth) ^b^	8
	Canada wildrye (*Elymus canadensis* L.) ^b^	8
	Little Bluestem (*Schizachyrium scoparium* (Michx.) Nash) ^d^	8
	Blue grama (*Bouteloua gracilis* (Willd. ex Kunth) Lag. ex Griffiths) ^d^	8
	Prairie sandreed (*Calamovilfa longifolia* (Hook.) Scribn.) ^d^	8

^a^ PLS = pure live seed; ^b^ Cool-season (C_3_) grass species; ^c^ Native legume species; ^d^ Warm-season (C_4_) grass species.

**Table 2:** Mean annual temperature and precipitation received during the experimental period in 2005–2014 and a long-term average (30 years) at the experimental site near Swift Current, SK, Canada.

Item	Months	2005	2006	2007	2008	2009	2010	2011	2012	2013	2014	30
Temperature (°C)	March	−1.0	−4.1	0.6	−2.3	−5.5	1.2	−8.1	1.7	−8.4	−6.5	−4.2
	April	6.4	8.6	4.7	2.9	4.2	6.1	2.8	5.5	−0.5	3.7	4.7
	May	9.7	12.3	11.6	10.8	10.0	8.2	9.5	9.9	12.7	11.0	10.9
	June	14.6	16.2	15.9	14.1	14.6	15.5	14.4	15.8	15.4	13.6	15.4
	July	18.3	21.2	22.9	17.8	17.0	17.1	18.3	20.0	17.1	18.2	18.7
	August	16.4	19.2	17.7	18.0	16.8	16.6	18.3	19.3	19.1	18.2	17.8
	September	12.2	12.7	11.7	12.3	17.0	10.9	15.4	14.2	15.4	12.5	12.0
	October	6.5	2.4	6.8	7.5	2.1	8.1	7.5	3.2	4.2	8.2	5.8
	November–February	−6.4	−5.4	−8.4	−8.8	−9.2	−9.4	−8.4	−4.8	−9.4	-8.8	−8.9
	Growing season (April–October)	12.0	13.2	13.0	11.9	11.7	11.8	12.3	12.6	11.9	12.2	12.2
	Grazing season (June–September)	15.4	17.3	17.1	15.6	16.4	15.0	16.6	17.3	16.8	15.6	16.0
	Annual	4.8	5.6	4.9	3.8	3.3	3.9	3.7	6.8	3.1	3.7	3.8
Precipitation (mm)	March	28.2	14.2	20.9	11.3	7.6	0.9	20.7	22.1	28.5	18.7	17.7
	April	26.0	19.4	18.6	17.5	15.2	51.4	31.8	42	25.3	39.1	21.3
	May	22.4	43.5	37.3	32.2	25.1	111.6	66.5	101.9	13.6	33.5	44.4
	June	123.2	99.9	56.0	142.7	37.8	126.3	116.9	113.4	113.0	116.0	74.7
	July	21.4	26.3	12.1	70.5	51.8	75.9	68.6	22.0	53.5	33.5	51.9
	August	52.1	24.1	23.4	71.8	61.3	95.8	35.7	10.9	17.6	105.6	43.5
	September	40.7	66.9	23.5	20.1	22.5	99.3	11.3	6.8	48.4	40.8	32.2
	October	9.4	15.8	14.0	13.8	27.6	9.7	37.2	21.2	6.4	17.3	18.8
	November-February	53.7	71.6	36.1	48.1	48.6	67.6	68.7	66.3	69.9	42.9	62.1
	Growing season (April–October)	295	296	185	369	241	570	368	318	278	386	287
	Grazing season (June–September)	237	217	115	305	173	397	233	153	233	296	202
	Annual	377	382	242	428	298	639	457	407	376	447	367

**Table 3:** The effect of seed mixture (7-mix, 12-mix), grazing system (continuous grazing (CG), deferred rotational grazing (DRG)) and production year (Yr) on pasture productivity and animal response.

Item ^a^	Maximum/Peak Forage DM Yield (kg ha^−1^) ^b^	Pre-Grazing Pasture DM Yield (kg ha^−1^)	Stocking Rate (AUM ha^−1^) ^c^	Grazing/Animal Days per ha ^d^	ADG (kg d^−1^)	TLW (kg ha^−1^) ^e^
Seed mixture (SM)						
7-mix	1493.7	1111.0	3.64	57.0	0.89	50.9
12-mix	1451.4	1126.5	3.69	62.7	0.91	57.8
SEM	98.8	87.5	0.290	3.6	0.071	3.68
Grazing system (GS)						
CG	1300.6 *^b^*	855.2 *^b^*	2.80 *^b^*	56.2 *^b^*	0.99 *^a^*	56.0
DRG	1644.4 *^a^*	1382.2 *^a^*	4.53 *^a^*	63.4 *^a^*	0.81 *^b^*	52.7
SEM	90.2	78.8	0.252	3.6	0.071	3.55
Year (Yr)						
2005	1861.7 *^a^*	981.6 *^ab^*	3.22 *^ab^*	69.0	0.62 *^b^*	34.0 *^b^*
2006	1214.2 *^bc^*	1153.5 *^a^*	3.78 *^ab^*	51.6	0.91 *^ab^*	47.7 *^bc^*
2007	1393.0 *^b^*	1069.4 *^a^*	3.51 *^ab^*	55.0	0.71 *^bc^*	38.9 *^b^*
2008	1600.8 *^a^*	1307.1 *^a^*	4.29 *^a^*	58.5	0.68 *^b^*	39.6 *^b^*
2009	1177.9 *^bc^*	1208.9 *^a^*	3.96 *^a^*	54.8	0.68 *^b^*	36.7 *^bd^*
2010	1723.8 *^a^*	1318.2 *^a^*	4.32 *^a^*	62.9	0.99 *^ac^*	63.5 *^acd^*
2011	1715.4 *^a^*	1308.6 *^a^*	4.29 *^a^*	72.0	1.04 *^a^*	72.7 *^a^*
2012	1719. 4 *^a^*	1322.4 *^a^*	4.32 *^a^*	55.2	1.01 *^a^*	69.6 *^ac^*
2013	1354.3 *^bc^*	737.2 *^b^*	2.42 *^b^*	54.9	1.14 *^a^*	62.1 *^ac^*
2014	964.8 *^c^*	780.4 *^b^*	2.56 *^b^*	64.2	1.19 *^a^*	79.0 *^a^*
SEM	174.1	141.0	0.462	5.8	0.114	8.24
Source of variance, *p*-Value						
SM	0.675	0.855	0.865	0.120	0.746	0.186
GS	0.0003	<0.0001	<0.0001	0.050	0.017	0.525
SM × GS	0.069	0.057	0.057	0.548	0.694	0.372
Yr	0.0001	0.002	0.002	0.186	0.001	0.0001
SM × Yr	0.946	0.978	0.978	0.999	0.985	0.998
GS × Yr	0.102	0.006	0.006	0.955	0.543	0.841
SM × GS × Yr	0.997	0.967	0.967	0.983	0.977	0.982

^a^ SEM = Standard error of the mean; ^b^ Maximum/peak yield was determined from the enclosure areas at the end of July; ^c^ AUM = animal unit month; calculated based on one 363 kg steer or the equivalent based upon average daily forage consumption of 10 kg dry matter (DM) [5]; ^d^ Grazing/animal days per ha = (total number of animals × number of days on test)/grazing area in ha; ^e^ TLW = Total live weight production was calculated as average daily gain (kg d^−1^) × grazing days per ha; *a*, *b*, *c*, *d* = Means within a column at each treatment and production year with different lower case letter are significantly different (*p* ≤ 0.05).

**Table 4:** The effect of seed mixture (7-mix, 12-mix), grazing system (continuous grazing (CG), deferred rotational grazing (DRG)) and production year (Yr) on pasture quality.

Item ^a^	OM (%)	OMD (%)	ADF (% DM)	NDF (% DM)	CP (% DM)	TDN (%) ^b^	DE (MJ kg^−1^ DM) ^c^	Total P (g kg^−1^ DM)
Seed mixture (SM)								
7-mix	89.9 *^b^*	52.4	34.9	58.3 *^b^*	6.77	59.5	10.9	1.70 *^a^*
12-mix	90.3 *^a^*	51.7	35.7	60.0 *^a^*	6.37	58.5	10.8	1.54 *^b^*
SEM	0.16	0.50	0.72	0.68	0.28	0.51	0.09	0.07
Grazing system (GS)								
CG	89.7 *^b^*	53.7 *^a^*	34.4 *^b^*	59.1	6.79	60.2 *^a^*	11.1 *^a^*	1.85 *^a^*
DRG	90.5 *^a^*	50.3 *^b^*	36.2 *^a^*	59.1	6.35	57.8 *^b^*	10.6 *^b^*	1.39 *^b^*
SEM	0.19	0.36	0.39	0.56	0.24	0.51	0.09	0.07
Year (Yr)								
2005	90.5 *^bf^*	47.1 *^c^*	35.7 *^ac^*	63.0 *^a^*	6.03 *^cd^*	58.4 *^bc^*	10.8 *^abc^*	1.83 *^a^*
2006	89.0 *^c^*	50.3 *^cd^*	34.1 *^bd^*	60.1 *^c^*	5.91 *^cd^*	60.6 *^a^*	11.1 *^a^*	1.71 *^ac^*
2007	88.9 *^c^*	51.3 *^bde^*	34.8 *^cd^*	61.0 *^ac^*	4.57 *^d^*	59.6 *^abc^*	11.0 *^a^*	1.25 *^bd^*
2008	89.6 *^cde^*	53.0 *^bde^*	34.9 *^bc^*	60.3 *^bc^*	6.59 *^c^*	59.5 *^abc^*	11.0 *^a^*	1.80 *^a^*
2009	88.9 *^cde^*	50.1 *^ce^*	34.8 *^bc^*	59.1 *^bc^*	5.21 *^d^*	59.6 *^abc^*	11.0 *^ab^*	1.13 *^d^*
2010	90.0 *^ef^*	54.4 *^ab^*	34.4 *^bc^*	60.4 *^bc^*	7.56 *^b^*	60.2 *^a^*	11.1 *^a^*	1.88 *^a^*
2011	90.7 *^ab^*	49.4 *^cde^*	36.2 *^a^*	60.7 *^ac^*	7.31 *^b^*	57.8 *^bc^*	10.6 *^bc^*	1.75 *^a^*
2012	91.4 *^a^*	50.1 *^cde^*	36.9 *^a^*	60.4 *^bc^*	6.61 *^c^*	57.0 *^c^*	10.5 *^b^*	1.71 *^a^*
2013	91.2 *^a^*	57.3 *^a^*	34.2 *^bc^*	55.8 *^d^*	7.28 *^b^*	60.5 *^abc^*	11.1 *^a^*	1.46 *^bc^*
2014	91.0 *^ab^*	57.5 *^a^*	37.0 *^a^*	50.6 *^d^*	8.65 *^a^*	56.8 *^c^*	10.5 *^c^*	1.67 *^ac^*
SEM	0.16	1.11	0.87	1.25	0.52	1.12	0.21	0.167
Source of variance, *p*-Value								
SM	0.035	0.334	0.066	0.015	0.162	0.065	0.061	0.027
GS	<0.0001	<0.0001	<0.0001	0.966	0.065	<0.0001	<0.0001	<0.0001
SM × GS	0.108	0.900	0.689	0.368	0.431	0.701	0.743	0.636
Yr	<0.0001	<0.0001	0.002	<0.0001	<0.0001	0.002	0.002	<0.0001
SM × Yr	0.996	0.995	0.367	0.024	0.787	0.368	0.341	0.718
GS × Yr	0.068	0.216	0.141	0.003	0.189	0.138	0.120	0.928
SM × GS × Yr	0.404	0.981	0.320	0.028	0.850	0.327	0.348	0.301

^a^ SEM = Standard error of the mean; ^b^ Total digestible nutrient (TDN, %) was calculated as 104.96 − (1.302 × acid detergent fiber (ADF) (% DM)) [48]; ^c^ DE (MJ kg^−1^ DM) = ((TDN, %/100) × 4.4 Mcal kg^−1^ TDN) × 4.184 MJ Mcal^−1^ [49]; *a*, *b*, *c*, *d*, *e*, *f* = Means within a column at each treatment and production year with different lower case letter are significantly different (*p* ≤ 0.05). Organic matter (OM), OM digestibility (OMD), crude protein (CP), neutral detergent fiber (NDF).

**Table 5:** Effect of seed mixture (SM, 7-mix, 12-mix), grazing system (GS, continuous grazing (CG), deferred rotational grazing (DRG)) and year (Yr) on pasture above ground biomass and litter carbon (C) and nitrogen (N) content (*n* = 80).

Item	Seed Mixture (SM)			Grazing System (GS)			Year (Yr)					Sources of Variance, *p*-Value ^a^			
	7-mix	12-mix	SEM ^b^	CG	DRG	SEM ^b^	2004	2008	2011	2014	SEM ^b^	SM	GS	SM × GS	Yr
Biomass (kg ha^−1^)	446	448	105.11	371	522	105.11	407 *^b^*	155 *^b^*	374 *^b^*	851 *^a^*	148.65	0.984	0.156	0.478	0.0003
C (kg ha^−1^)	178	183	43.05	147	214	43.05	156 *^b^*	61 *^b^*	156 *^b^*	350 *^a^*	60.89	0.913	0.125	0.432	0.0002
N (kg ha^−1^)	2.93	2.73	0.59	2.58	3.08	0.59	3.43 *^a^*	0.93 *^b^*	2.35 *^ab^*	4.61 *^a^*	0.83	0.732	0.394	0.757	0.001
C:N ratio	61.8	65.7	4.32	59.3	68.2	4.32	45.4 *^b^*	66.1 *^a^*	67.0 *^a^*	76.5 *^a^*	6.12	0.378	0.045	0.233	<0.0001
Litter (kg ha^−1^)	218	226	27.40	188 *^b^*	256 *^a^*	27.40	329 *^a^*	224 *^b^*	116 *^c^*	219 *^b^*	38.76	0.796	0.018	0.717	<0.0001
C (kg ha^−1^)	81.4	82.2	14.32	67.94 *^b^*	95.68 *^a^*	11.29	124.01 *^a^*	71.89 *^b^*	44.70 *^c^*	86.63 *^b^*	16.11	0.953	0.018	0.870	0.0001
N (kg ha^−1^)	1.50	1.60	0.18	1.34 *^b^*	1.75 *^a^*	0.18	2.16 *^a^*	1.67 *^a^*	0.86 *^c^*	1.50 *^bc^*	0.25	0.595	0.031	0.826	<0.0001
C:N ratio	53.7	51.8	2.40	51.8	53.7	2.12	58.2 *^a^*	43.1 *^c^*	52.0 *^b^*	57.6 *^ab^*	3.04	0.431	0.366	0.408	<0.0001

^a^ The interaction effects for SM × Yr, GS × Yr and SM × GS × Yr were not significant for all variables (*p* > 0.05), except for litter N (kg ha^−1^) with SM × GS × Yr (*p* = 0.05), and values were not included in the table; ^b^ SEM = Standard error of the mean; *a*, *b*, *c* = Means within a row at each treatment and production year with different lower case letter are significantly different (*p* ≤ 0.05).

**Table 6:** Effect of seed mixture (SM, 7-mix, 12-mix) × grazing system (GS, continuous grazing (CG), deferred rotational grazing (DRG)) interaction and year (Yr) on soil properties (carbon (C), C to nitrogen (N) ratio, bulk density, moisture, equivalent soil mass) sampled at 0-15 and 0-30 (0-15 plus 15-30) cm depth (*n* = 120).

Item	7-mix		12-mix		SEM ^b^	Year (Yr)					SEM ^b^	Sources of Variance, *p*-Value ^a^			
	CG	DRG	CG	DRG		2000	2004	2008	2011	2014		SM	GS	SM × GS	Yr
Soil (15 cm depth)															
Organic C (Mg ha^−1^)	24.45 *^c^*	30.46 *^ab^*	31.00 *^a^*	27.78 *^b^*	1.82	27.61 *^b^*	30.97 *^a^*	26.74 *^b^*	25.97 *^b^*	30.81 *^a^*	1.85	0.071	0.190	0.001	0.005
C:N ratio	9.53	9.60	9.83	9.53	0.16	9.14 *^b^*	9.24 *^b^*	8.99 *^b^*	8.57 *^c^*	12.16 *^a^*	0.20	0.400	0.379	0.174	<0.0001
BD (g cm^−3^)	1.56	1.54	1.56	1.54	0.03	1.65 *^a^*	1.61 *^a^*	1.41 *^d^*	1.52 *^c^*	1.55 *^b^*	0.03	0.763	0.261	0.952	<0.0001
Moisture (%)	16.76 *^a^*	14.96 *^b^*	14.79 *^b^*	14.99 *^b^*	0.56	17.30 *^a^*	16.40 *^ab^*	16.47 *^a^*	11.55 *^c^*	15.14 *^b^*	0.72	0.036	0.083	0.031	<0.0001
Soil (30 cm depth)															
Organic C (Mg ha^−1^)	42.33 *^b^*	48.42 *^a^*	49.07 *^a^*	46.29 *^ab^*	1.80	46.59 *^bc^*	48.67 *^ab^*	43.11 *^c^*	42.85 *^c^*	51.42 *^a^*	2.33	0.123	0.267	0.004	0.002
C:N ratio	9.26	9.31	9.52	9.20	0.18	8.59 *^bc^*	8.84 *^b^*	8.63 *^b^*	8.28 *^c^*	12.28 *^a^*	0.23	0.611	0.383	0.207	<0.0001
BD (g cm^−3^)	1.54	1.51	1.51	1.54	0.02	1.63 *^a^*	1.58 *^b^*	1.39 *^d^*	1.48 *^c^*	1.55 *^b^*	0.023	0.739	0.941	0.057	<0.0001
Moisture (%)	15.91	14.66	14.48	14.36	0.46	14.89 *^b^*	16.21 *^a^*	15.15 *^ab^*	11.79 *^c^*	16.22 *^a^*	0.59	0.026	0.075	0.135	<0.0001
Equivalent soil mass (*n* = 120) ^c^															
Organic C (Mg ha^−1^)	37.22 *^b^*	44.19 *^a^*	44.77 *^a^*	41.17 *^ab^*	2.28	39.24 *^b^*	43.33 *^ab^*	41.56 *^ab^*	39.58 *^b^*	45.48 *^a^*	2.47	0.126	0.247	0.012	0.040
C:N ratio	9.25	9.33	9.58	9.23	0.17	8.83 *^b^*	8.87 *^b^*	8.58 *^bc^*	8.23 *^c^*	12.23 *^a^*	0.22	0.405	0.330	0.131	<0.0001

^a^ The interaction effects for SM × Yr, GS × Yr and SM × GS × Yr were not significant for all variables (*p* > 0.05), except for soil C:N (0-15 cm) with GS × Yr (*p* < 0.0001), soil moisture (0–15 cm) with GS × Yr (*p* = 0.05), and soil C:N (0–30 cm) with GS × Yr (*p* = 0.001), and the values were not included in the table; ^b^ SEM = Standard error of the mean; ^c^ Equivalent soil mass of 4000 Mg ha^−1^ was used for calculation (0–30 cm sampling depth) following Ellert and Bettany [45] and Ellert et al. [46]; *a*, *b*, *c* = Means within a row at each treatment and production year with different lower case letter are significantly different (*p* ≤ 0.05).

## Appendix A

**Table A1:** Foliar cover (%) of seeded native and other species for the 7- and 12-mix pasture in 2004 and 2014.

Item	2004		2014	
	7-Species (7-mix)	12-Species (12-mix)	7-Species (7-mix)	12-Species (12-mix)
Western wheatgrass (*Pascopyrum smithii* (Rydb.) Barkworth & D.R.) ^a^	11.9	7.6	4.9	1.5
Northern wheatgrass (*Elymus macrourus* (Turcz. *ex* Steud.) Tzvelev) ^a^	8.4	8.5	5.3	1.6
Awned wheatgrass (*Elymus trachycaulus* (Link) Gould & Shin. Ssp *subsecundus* (Link) A.&D. Love.) ^a^	3.9	2.9	1.3	0.2
Slender wheatgrass (*Elymus trachycaulus* (Link) Gould subsp. *Trachycaulus*) ^a^	9.9	4.6	0.1	0.0
June grass (*Koeleria macrantha* (Ledeb.) Schult.) ^a^	0.2	0.3	3.9	1.9
Green needle grass (*Nassella viridula* (Trin.) Barkworth) ^a^	0.3	2.2	2.4	0.5
Purple prairie clover [*Dalea purpurea* Vent.) ^b^	0.03	0.7	5.6	8.8
Needle and thread grass (*Hesperostipa comata* (Trin. & Rupr.) Barkworth) ^a^	0.0	0.2	0.0	0.3
Canada wildrye (*Elymus canadensis* L.) ^a^	0.01	0.01	0.0	0.0
Little Bluestem (*Schizachyrium scoparium* (Michx.) Nash) ^c^	0.01	6.3	0.3	24.8
Blue grama (*Bouteloua gracilis* (Willd. ex Kunth) Lag. ex Griffiths) ^c^	0.3	1.9	0.0	2.9
Prairie sandreed (*Calamovilfa longifolia* (Hook.) Scribn.) ^c^	0.0	0.4	0.0	0.6
Foliar cover of seeded native species	34.95	35.61	23.80	43.10
Foliar cover of other ^d^	65.05	64.39	76.20	56.90

^a^ Cool-season grasses (C_3_); ^b^ Native legume species; ^c^ Warm-season grasses (C_4_); ^d^ Other foliar cover included moss, litter, weed (all introduced species including other native species that was not part of seeded mix), bare ground and rock/dung.
